# Supplementary figures and images for: Using the Bayesian credible subgroups method to identify populations benefiting from treatment: An application to the Look AHEAD trial
Source: PLoS One. 2020 Apr 21;15(4):e0231241. doi: 10.1371/journal.pone.0231241 (PMC7173866; doi:10.1371/journal.pone.0231241)

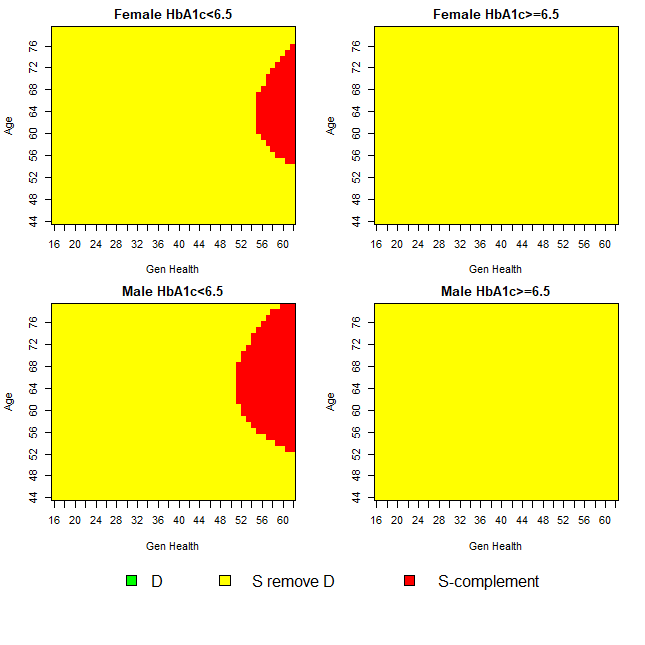

Supplement: S1 Fig — Allows for assumptions of treatment effect homogeneity within all levels of selected covariates. (TIF) [file pone.0231241.s001.tif]

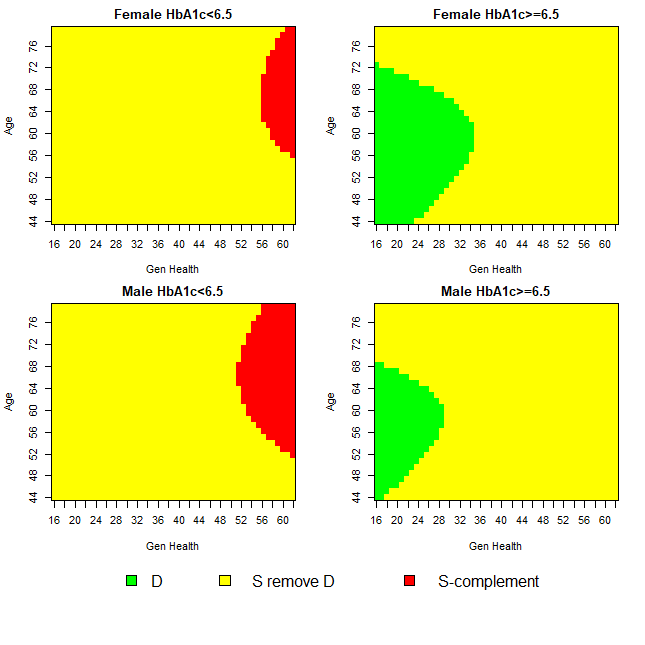

Supplement: S2 Fig — Individual credible subgroup pairs plotted over the covariate space. There is at least 65% posterior probability that all patients with covariate points in D have a conditional average treatment effect positive treatment effect (δ = 0). (TIF) [file pone.0231241.s002.tif]

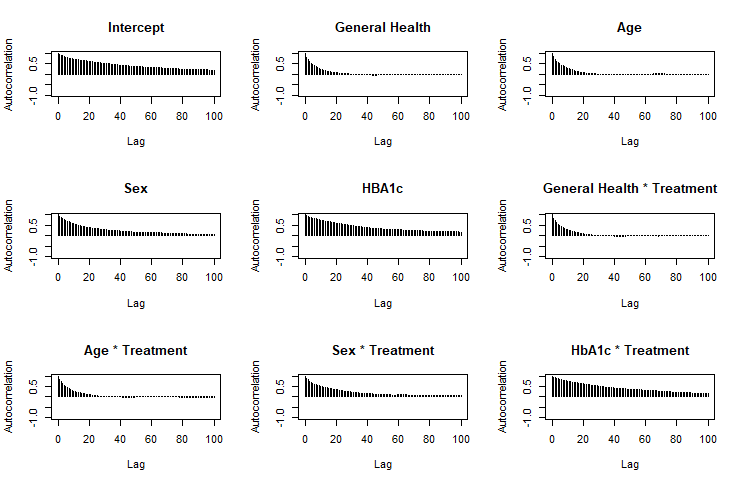

Supplement: S3 Fig — (TIF) [file pone.0231241.s003.tif]

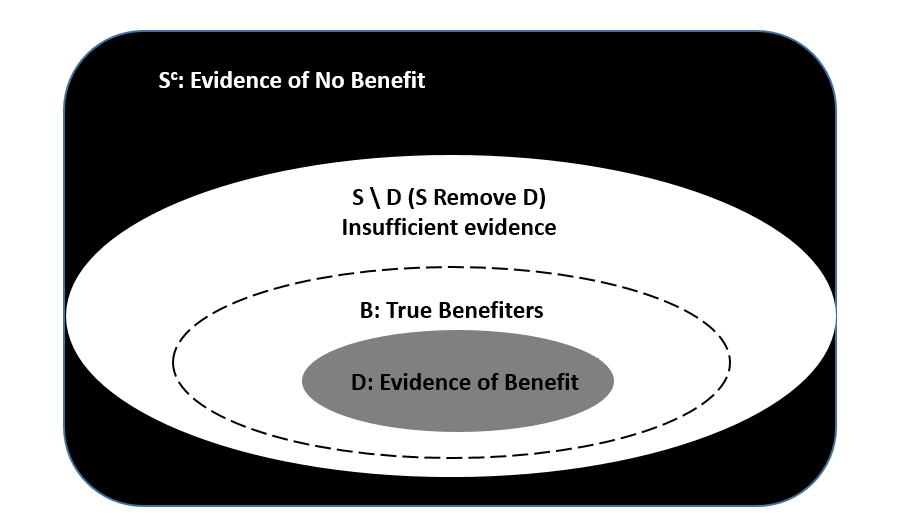

Supplement: S4 Fig — Visualisation of the trichotomy of the covariate space based on the credible subgroup pair (D,S) relative to the true benefiting subgroup (B), adapted from Schnell et.al. [6] (TIF) [file pone.0231241.s004.tif]

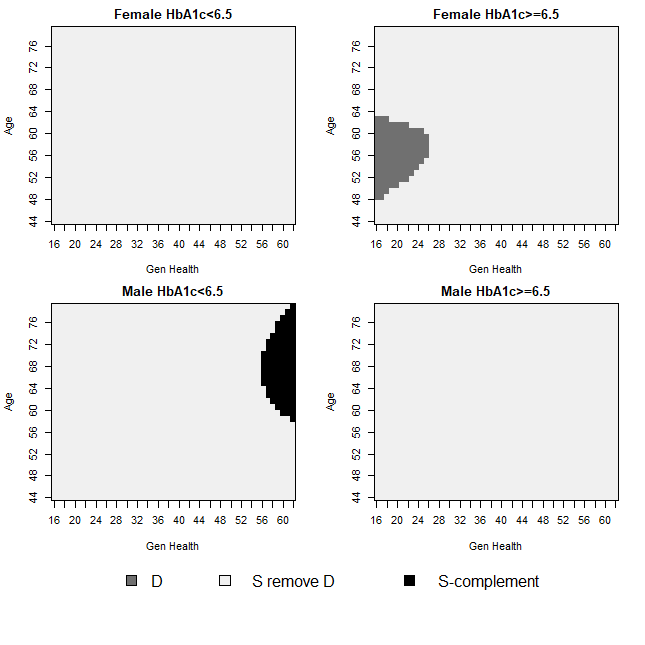

Supplement: S5 Fig — Individual credible subgroup pairs plotted over the covariate space. There is at least 80% posterior probability that all patients with covariate points in D have a positive conditional average treatment effect (δ = 0). (TIF) [file pone.0231241.s005.tif]

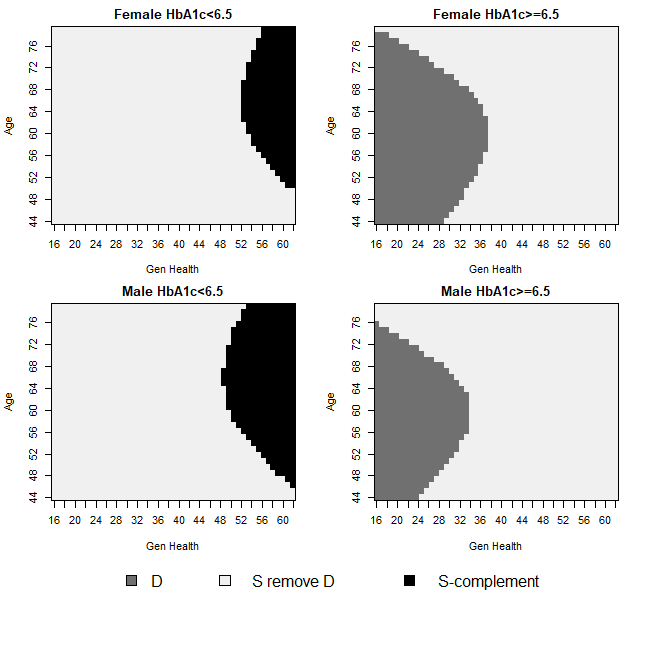

Supplement: S6 Fig — Individual credible subgroup pairs plotted over the covariate space. There is at least 50% posterior probability that all patients with covariate points in D have a positive conditional average treatment effect treatment effect (δ = 0). (TIF) [file pone.0231241.s006.tif]

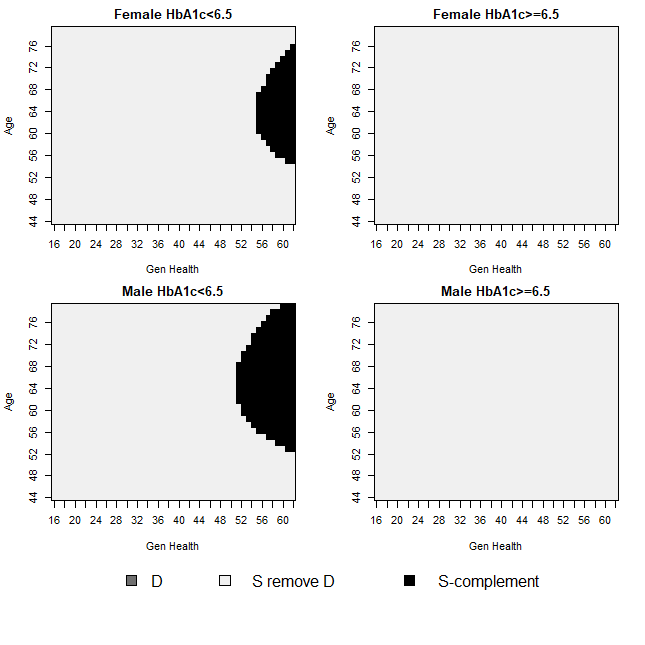

Supplement: S7 Fig — Allows for assumptions of treatment effect homogeneity within all levels of selected covariates. (TIF) [file pone.0231241.s007.tif]

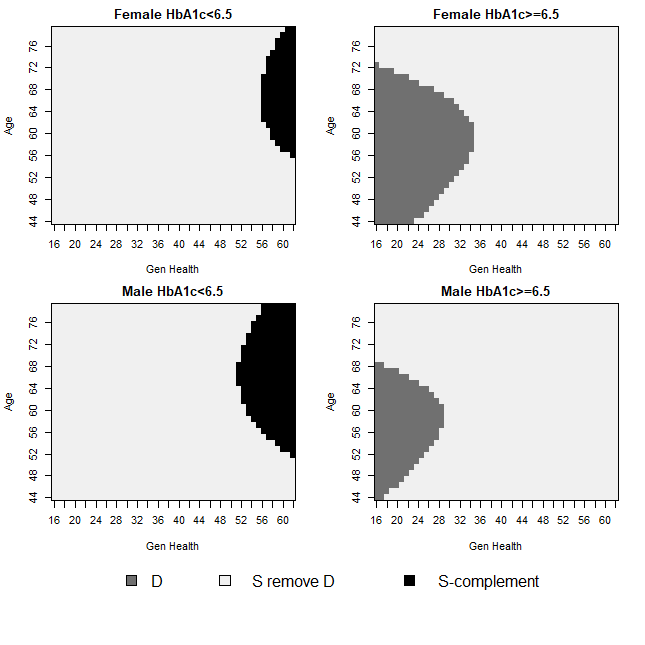

Supplement: S8 Fig — Individual credible subgroup pairs plotted over the covariate space. There is at least 65% posterior probability that all patients with covariate points in D have a conditional average treatment effect positive treatment effect (δ = 0). (TIF) [file pone.0231241.s008.tif]
